# Low Infrared Emissivity and Strong Stealth of Ti-Based MXenes

**DOI:** 10.34133/2022/9892628

**Published:** 2022-05-23

**Authors:** Xinliang Li, Minghang Li, Xin Li, Xiaomeng Fan, Chunyi Zhi

**Affiliations:** ^1^Department of Materials Science and Engineering, City University of Hong Kong, 83 Tat Chee Avenue, Kowloon, Hong Kong 999077, China; ^2^Science and Technology on Thermostructural Composite Materials Laboratory, Northwestern Polytechnical University, Xi'an 710072, China

## Abstract

Advanced scenario-adaptable infrared (IR) stealth materials are crucial for creating localized closed thermal environments. Low emissivity over the broadest possible band is expected, as is superior mechanical deformability. Herein, we report a series of Ti-based MXenes with naturally low emissivity as ideal IR shielding materials. Over a wavelength ranging from 2.5 to 25 *μ*m, Ti_3_C_2_T_*X*_ film delivers an average emissivity of 0.057 with the lowest point of 0.042. Such a low emissivity coupled with outstanding structural shaping capability is beyond the current grasp. The reflection-dominated mechanism is dissected. Also, some intriguing scenarios of IR stealth for wearable electronic devices and skin thermal control are demonstrated. This finding lights an encouraging path toward next-generation IR shielding by the expanding MXene family.

## 1. Introduction

Any object above absolute zero degrees constantly emits radiation, primarily in the infrared (IR) range [1, 2]. Customizing adaptive thermal environments is therefore of great significance for scientific research, commercial industry, or even military camouflage [3–5]. Following this, considerable efforts have been devoted to regulating IR/thermal radiation and achieving desired heat isolation and IR stealth in time-varying surroundings. The Stefan-Boltzmann theorem (*E* = *εσT*^4^) concludes that the IR emissivity (*ε*) is a linear function of the IR radiation intensity of the heat source [6, 7]. Therefore, at a given temperature (*T*), reducing the IR emissivity of protected targets to elude the perception of the IR detector should be the most effective [8]. As a result, materials with low IR emissivity, which will not dissipate the heat in radiating manner, are preferred [9, 10]. Also, wide applicable bandwidth is expected to benefit the practical scenarios. Currently, a fraudulent coating is the most effective way to achieve these since the IR radiation is mainly determined by the shallow surface.

Unfortunately, naturally low emissivity materials are scarce [11]. The most mature polished metals possess decent performance, but even slight surficial roughness or oxidation will cause severe variation in the emissivity by several times [12]. Besides, stringent processing requirements are inevitable to match the irregular structure of the targets. Another promising alternative configuration refers to the composite with fillers of polar oxides [13], metamaterials [14], carbon [15], conjugate polymers [16], photonic crystal [17], and phase-change materials [18]. As a matter of fact, they are normally macroscopically discontinuous and necessitate external binders or film shapes to achieve structural shaping, whereas the high emissivity additives tend to offset their intrinsic superiority [19]. Due to the lack of flexibility, their application may be not fit for some next-generation application scenarios, such as wearable smart textiles and flexible energy storage devices [20, 21]. Therefore, predictable rigorous demands call for breakthroughs.

Transition metal carbide/nitrides, with a general expression of *M*_*n*+1_*X*_*n*_*T*_*X*_ (*M*: transition metal atoms; *X*: C or/and N; *T*_*X*_: surficial terminations; *n*: atom layer number), denoted as MXene, were recruited into a two-dimensional (2D) family in 2011 [22]. The past decade has witnessed their incredible prosperity relying on phase abundance, high electrical conductivity, and customized physiochemical properties [23]. Mature synthesis processes can fabricate more than 30 kinds of MXenes. They excel in mechanical strength, electrochemistry, biomedicine, sensor, catalysis, thermoelectricity, microwave absorption/shielding, and composite materials [24–27]. Focusing on the optical field, plasmonic properties, optical detection, and photothermal effects were investigated in depth, in which both surface terminations and body elements carry considerable weight [28]. Despite this, the fundamental IR properties and emissivity are still in the shadows to date.

## 2. Results

In this contribution, we pioneered the IR properties of MXenes and reported their unparalleled low IR emissivity over a broad wavelength range of 2.5-25 *μ*m. The direct active blackbody radiation source method (2-20 *μ*m) and indirect Fourier transform infrared spectroscopy (FTIR) technique together revealed that the IR emissivity of Ti_3_C_2_T_*X*_ film at only 300 nm is distributed in the range of 0.042-0.140, with an average value of 0.057. Furthermore, remarkable universality is recognized in representative 211-type Ti_2_CT_*X*_ MXene and solid-solution TiVCT_*X*_ MXene, declaring absolute dominance against synthetic IR stealth materials. In addition, with excellent chemical affinity to both organics and inorganics, MXene flakes can also act as effective emissivity regulators with structure shapeable function for customizing IR stealth composites with the desired emissivity. As a validation, we demonstrated the impressive IR stealth of MXenes on a wearable energy storage capacitor. In our design, the IR emissivity of targets was effectively reduced by either simple overlaying or microscopic wrapping.

**Figure 1:**
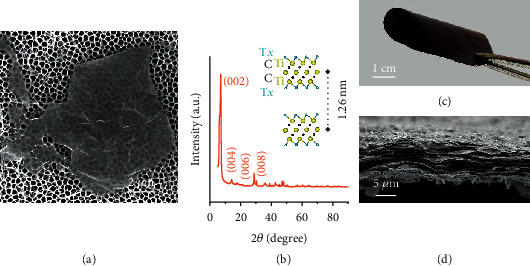
Synthesis and characterization of Ti_3_C_2_T_*X*_ MXene and film. (a) SEM image of a Ti_3_C_2_T_*X*_ MXene flake. (b) XRD pattern of pristine Ti_3_C_2_T_*X*_ MXene. Inset illustrates the crystal structure, where the yellow ball represents the Ti atom, black ball represents C atom, and blue ball represents the surficial terminations. (c) Digital photography of the Ti_3_C_2_T_*X*_ MXene film with superior mechanical flexibility. (d) Cross-sectional SEM image of Ti_3_C_2_T_*X*_ MXene film.

Ti_3_C_2_T_*X*_ MXene flakes were produced *via* a typical wet chemical path with Ti_3_AlC_2_ MAX parent and HCl+LiF etchant (see Methods for details). A scanning electron microscope (SEM) image shows that the resultant Ti_3_C_2_T_*X*_ MXene flake holds a lateral size of several microns (Figure 1(a)). No visible impurities can be detected on the smooth surface. Also, the electron beam transparency feature is identified, indicating the flake's thickness at the nanometer level. As noteworthy are the surficial wrinkles and folding at the upper edge resulting from the superior flexibility. The X-ray diffraction pattern (XRD) further characterizes the phase composition where the departure of Al atom layers leads to the absence of diffraction peak of (104) crystal plane at about 39° (Figure 1(b)) [29]. The subsequently terminated surface termination (=O, -OH, -Cl, -F) further expands the interlayer spacing. Accordingly, the diffraction peak indexed to (002) basal plane gets blue-shifted to 7.04°, corresponding to a ca. 1.26 nm spacing, as depicted in the inset. Moreover, this sharp peak with a narrow half-width and vigorous relative intensity evidences the prominent crystallization of Ti_3_C_2_T_*X*_ MXene.

With their large and thin nature, as well as the functional surface, Ti_3_C_2_T_*X*_ MXene flakes are readily film-forming through vacuum filtration. Wan der Waals force and hydrogen bonds bind the dispersed flakes tightly to form a self-standing macroscopic film [30]. The excellent flexibility allows it to withstand mechanical deformation without cracking, such as bending, as demonstrated in Figure 1(c). The cross-sectional microscopic characterization reveals the fact that the Ti_3_C_2_T_*X*_ MXene flakes are densely stacked (Figure 1(d)). The close-knit character contributes to the impressive mechanical performance and diminishes the surficial roughness. Also, the atomic force microscope (AFM) image reveals the lamination arrangement of Ti_3_C_2_T_*X*_ MXene flakes with extremely small fluctuation, as shown in Extended Data Figure 1.

We then perform an infrared emissivity test to directly characterize the IR emissivity of Ti_3_C_2_T_*X*_ MXene within the wavelength range of 2-20 *μ*m under ambient conditions. Note that the as-prepared fresh MXene films with different thicknesses are employed as the research objects, and the hemispherical blackbody probe is vertically covered on its surface. For comparison, we also measure many other representative materials, including carbon, oxides, polymers, wood, composites, and polished metal foils.

**Figure 2:**
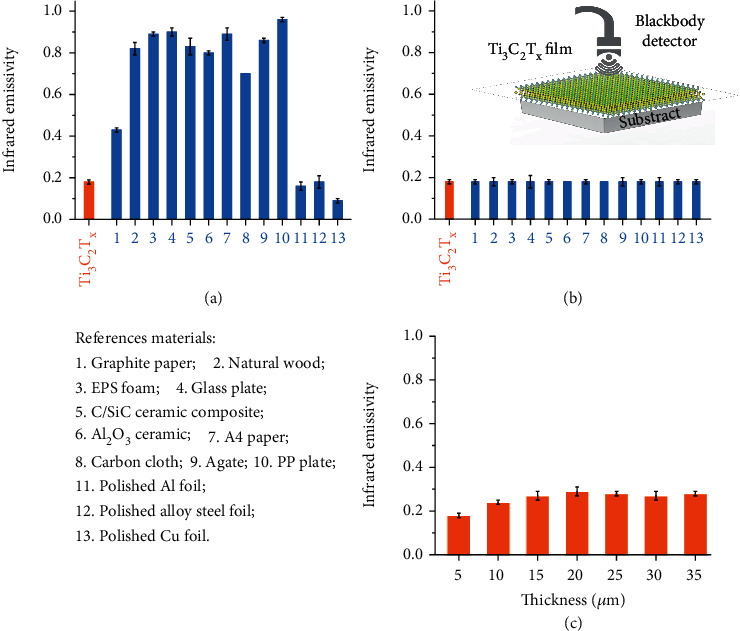
IR emissivity characterization with direct active blackbody radiation source method over a wavelength of 2-20 *μ*m under ambient conditions. (a) IR emissivity of Ti_3_C_2_T_*X*_ MXene film with 5 *μ*m thickness and representative reference materials. The material numbers correspond to the notes shown in the box below. (b) IR emissivity of abovementioned reference materials as substrates being protected and MXene film with 5 *μ*m thickness. Inset illustrates the models of composite structure and test mode. (c) IR emissivity of Ti_3_C_2_T_*X*_ MXene films with different thicknesses.

The IR emissivity of Ti_3_C_2_T_*X*_ MXene film with a thickness of 5 *μ*m is estimated to be 0.18 ± 0.01, which is undoubtedly an impressively low value among synthetic and natural materials. The IR emissivity of pure nonmetallic materials can hardly be lower than 0.4 over a broad wavelength range, even at favorable high-temperature conditions [31]. The values of all nonmetallic samples are much higher than that of MXene film, and PP (polypropylene) plate possesses a maximum number reaching 0.96 ± 0.02, as summarized in Figure 2(a). Thus, Ti_3_C_2_T_*X*_ MXene is concluded to be an inherently low IR emissivity material comparable to metal.

Additionally, we further explore the IR stealth performance of MXene. We used the abovementioned reference materials as substrates protected by MXene film shelter over the signal reception area, as illustrated in the inset in Figure 2(b) and Extended Data Figure 2. Experimental results record a striking emissivity shift for both low emissivity metal and high emissivity nonmetal samples. All values measured are close to 0.18 of the free-standing Ti_3_C_2_T_*X*_ MXene film, indicating that the 5 *μ*m thick MXene shelter is capable of fully shielding the IR radiation of substrates (Figure 2(b)). The thermal radiation energy that transmits is minimal. In short, a thin MXene shelter can effectively block IR radiation and create thermal environments that isolate surroundings.

Generally, for the electromagnetic interference (EMI) materials, including Ti_3_C_2_T_*X*_ MXene, the EMI effectiveness (SE) is positively correlated with the thickness; that is, simply increasing the thickness can enhance the EMI SE [25]. To explore whether this effect holds for the IR region, we measure the IR emissivity of Ti_3_C_2_T_*X*_ MXene films with different thicknesses. As shown in Figure 2(c), the IR emissivity initially increases with elevating thickness and then stabilizes; they are 0.24 ± 0.01 for 10 *μ*m, 0.27 ± 0.02 for 15 *μ*m, 0.29 ± 0.02 for 20 *μ*m, 0.28 ± 0.01 for 25 *μ*m, 0.27 ± 0.02 for 30 *μ*m, and 0.28 ± 0.01 for 35 *μ*m. This can be explained that with the increased amount of absorber content, the ability to absorb incident IR waves gets strengthened, which subsequently boosts the IR emissivity, similar to the evolution of EMI SE from absorption in EMI SE [25, 32].

**Figure 3:**
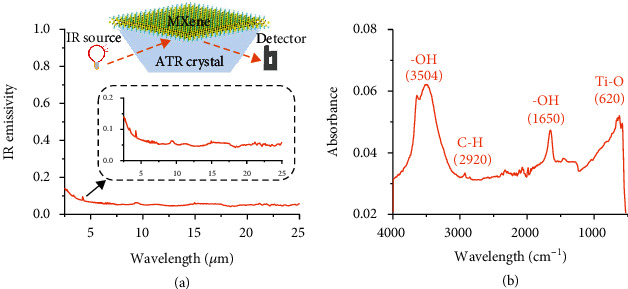
IR emissivity characterization with FTIR spectroscopy over a wavelength of 2.5-25 *μ*m at room temperature. (a) The IR emissivity versus wavelength profile of a Ti_3_C_2_T_*X*_ MXene film with 300 nm thick. Inset indicates the data curve details and illustrates the test mode. (b) The FTIR-ATR spectroscopy of Ti_3_C_2_T_*X*_ MXene film with 5 *μ*m thickness.

Subsequently, FTIR spectroscopy was implemented to build up a precise correlation between wavelength and IR emissivity for Ti_3_C_2_T_*X*_ MXene film in an IR region ranging from 4000 cm^−1^ (2.5 *μ*m) to 400 cm^−1^ (25 *μ*m). We can get the absorbance for opaque objects by subtracting the measured reflectivity from 1 [18]. Note that based on the Kirchhoff law [3], the radiation energy absorbed by an object denoted as absorbance equals the IR emissivity at thermodynamic equilibrium. As shown in Figure 3(a), within the whole IR wave range, the emissivity of Ti_3_C_2_T_*X*_ MXene film coated on the glass surface with a thickness of about 300 nm is distributed within 0.042 to 0.140, resulting in an average value of 0.057. Such a low value is extremely rare for reported synthetic materials.

Additionally, FTIR spectroscopy on the attenuated total reflectance (FTIR-ATR) mode was further collected to clarify the unavoidable interaction between surficial terminations and incident IR waves. Figure 3(b) exhibits the profile of absorbance versus wavelength. Three prominent peaks are detected, and they are attributed to chemical bond vibrations [28]. To be specific, hydroxyl groups as surface-bonded functional groups, interlayer intercalated water molecules, or externally adsorbed water molecules trigger the absorption peaks at 3504 and 1650 cm^−1^. The peak at 620 cm^−1^ comes from the deformation vibration of the Ti-O bonds, and the one at 2920 cm^−1^ is assigned to C-H bonds. Thus, we reasonably speculate that the naturally functionalized surface enhances the absorption ability of MXene film to IR waves in these specific regions and hence increases the IR emissivity. This may inspire future research on manipulating the IR emissivity of MXenes.

To demonstrate the practical IR stealth capabilities of MXene covering, we design intriguing and cutting-edge scenarios of wearable energy storage devices and skin temperature regulation. An IR thermography monitor is employed to visualize the thermal radiation of target objects, including flexible electrodes, wearable quasisolid capacitors, wearable textile, and skin.

**Figure 4:**
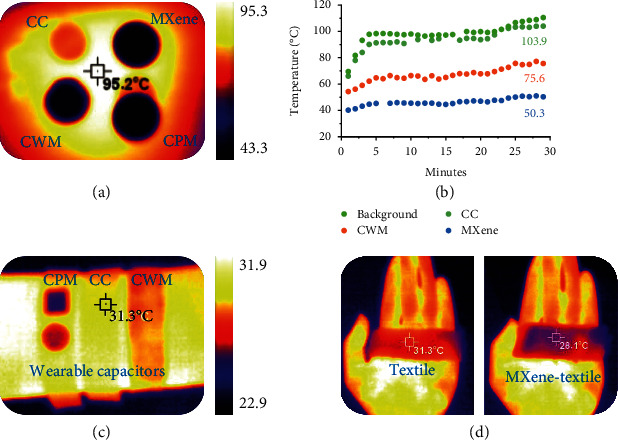
Demonstrations of IR stealth and skin thermal regulation. (a) IR image of CC, CWM, CPM, and MXene film placed on a hot plate. (b) The dynamic time-temperature curve of the four samples. (c) IR images of flexible quasisolid capacitors based on CC, CPM, and CWM electrodes worn on the wrist. (d) IR images of skin covered by commercial textile and MXene-functionalized textile.

For flexible electrode demonstration, four samples are prepared (Extended Data Figure 3): sample 1 is a carbon cloth (CC) current collector, sample 2 is pure MXene film, sample 3 is the carbon cloth physically half-covered with MXene film (CPM), and sample 4 is the MXene-wrapped carbon cloth obtained by a facile self-assembly process (CWM; Extended Data Figure 4). As displayed in Figure 4(a), four samples placed on a thermostatic hot plate present different radiation states in the IR mode. Intuitively, after 120 seconds of heating, the lowest temperature (43.3°C) is obtained at the MXene covered part of the CPM, where the IR signal is entirely masked. The temperature of the exposed part without MXene covering is close to CC (90.1°C vs. 91.4°C). The other three samples follow a temperature sequence of CC (90.1°C) > CWM (62.2°C) > MXene (44.7°C), which firmly verifies the effective IR stealth performance of MXenes. Furthermore, refined dynamic time-temperature curves and thermal images (Extended Data Figure 5) are recorded over thirty minutes. Within about 30 s, the temperature of the CC sample rises sharply to about 78.5°C, and eventually, its IR signature ultimately merged with the background. In stark contrast, the temperature of MXene films at 30 s is only 41.2°C and remains within 50.3°C for the subsequent test (103.9°C for CC). The CWM shows a moderate evolution, as shown in Figure 4(b). These results indicate that the introduced MXene can shield the IR emission from the position they cover, thus showing a lower temperature.

To demonstrate the IR stealth of MXene to wearable electronic devices, we constructed a flexible quasisolid capacitor based on the above CC, CPM, and CWM electrodes (see Extended Data Figure 6 and Methods for details). A similar IR law is observed at room temperature; that is, the CC-based capacitor worn on the wrist releases the strongest IR signal, followed by CWM, as shown in Figure 4(c). The MXene ornaments (circle and rectangle) on the CPM counterpart entirely mask the IR signature. Therefore, the MXene flakes or interface layer effectively camouflages the covered region and makes it invisible to the IR detector.

After validating the excellent IR shielding property with low emissivity, we confirm that MXene plays a significant role in stabilizing skin thermal regulation [20]. By simply soaking in an aqueous MXene solution, the commercial textile is equipped with the IR blocking function without damaging the flexibility (Extended Data Figure 7). As demonstrated in Figure 4(d), the skin region covered by the pristine textile products possesses intense IR radiation. In contrast, the MXene-functionalized one makes the covered skin perceive lower temperature (28.1°C versus 31.3°C). Based on our results, two intriguing application scenarios can be proposed: in high-temperature environments, the MXene-functionalized textile can stabilize skin temperature by blocking IR radiation from the external surroundings; on the contrary, under low-temperature conditions, it can prevent skin radiation from leaking to the outside and maintain the localized perceived temperature to protect the skin.

Customizable composition and atom arrangement make MXenes diversified. To validate the universality of low emissivity character, we further explore the 211-type Ti_2_CT_*X*_ MXene and solid-solution TiVCT_*X*_ MXene. The 5 *μ*m thick Ti_2_CT_*X*_ MXene film shows an IR emissivity of 0.22 ± 0.01 under the ambient condition. For solid-solution TiVCT_*X*_ MXene, the IR emissivity is calculated to be 0.20 ± 0.01. These results verify that 312 and 211 types of Ti-based MXenes are naturally low emissivity materials. The exploration of other MXene films is pending an upgrade to the synthesis process [33, 34].

**Figure 5:**
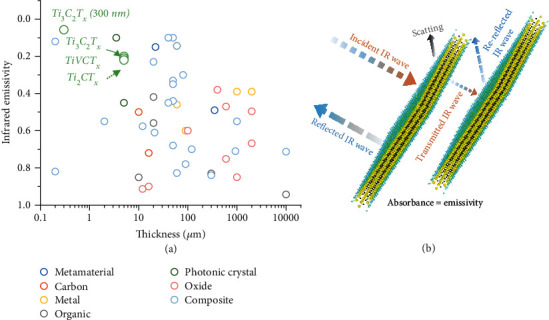
Universality, superiority, and mechanism analysis. (a) IR emissivity comparison of this work with reported materials, including metamaterials (blue circle), polished metal (gold circle), organic (grey circle), photonic crystal (green circle), oxide (rosy circle), and composite (wathet circle). Details are given in Extended Data Table 1. (b) Schematic illustration of the proposed low IR emissivity and stealth mechanisms of MXenes.

Of great concern is that the low emissivity of MXenes is superior to most reported peers after extensive literature review, including metamaterial, metal, carbon, oxides, polymer, photonic crystal, and composites, as compared in Figure 5(a) and Extended Data Table 1. Taking their appealing structuring, machinability, and easy-to-composite features into account, which are highly favored for commercial scale-up, MXenes and their composites are foreseeable to be more scenario-adaptable, leading the next generation of advanced IR stealth products.

We then propose the fundamental IR stealth mechanism of MXenes, as illustrated in Figure 5(b). The low IR emissivity is the result of high reflectivity. According to the Hagen-Rubens theorem, the electrical conductivity of an object is essentially synonymous with the reflection coefficient in the IR range [35]. As the IR waves hit the exposed surface of the MXene, the majority of energy is reflected directly and cannot reach the inside because the metallic MXene is extremely conductive [30, 36]. Then, the survived IR waves also suffer from rereflection by the inner flakes. In addition, the compact stacked flexible nanothick flakes reduce the surface roughness, which is beneficial to enhance the normal reflection while weakening the probability of IR wave absorption caused by the scatting behavior [10]. For the surface-functionalized MXenes, the presence of terminated groups, however, will intensify the interaction with internal IR waves, thus increasing the absorption coefficient and emissivity. This effect is recognized as beneficial in the microwave band (8-12.4 GHz) [25], but the opposite is valid for IR waves. Note that other experimental studies and density functional theory (DFT) simulations show the plasmonic resonance and interband transitions also the optical response of MXenes [36].

## 3. Discussion

In conclusion, we have pioneered the low IR emissivity and excellent IR stealth function of MXene with micrometer thick over a broad IR range (2.5-25 *μ*m) *via* the blackbody radiation source and FTIR spectroscopy measures. The mechanism concerning dominated reflection, detrimental adsorption, and scatting is analyzed anatomically. Also, the universality of this attractive attribute of other Ti-based MXenes is validated. Such a low emissivity of 0.042 coupled with exceptional machinability makes MXenes outperform the current grasp and lead the advance of the next generation of IR shielding. Furthermore, we have visualized some intriguing practical scenarios of IR stealth for wearable electronic devices and skin thermal regulation. Our discovery discloses a novel and unparalleled IR property of the MXene family, which may pique the interest of MXene, optical, and interdisciplinary communities.

## Data Availability

All data needed to evaluate the conclusions in the paper are present in the paper and/or Supplementary Materials. Additional data related to this paper may be requested from the authors.

## Supplementary Materials

Supplementary Materials Extended Data Figure 1: AFM image of Ti_3_C_2_T_*X*_ film. Extended Data Figure 2: digital images of the reference samples that are physically covered by MXene film. The numbers correspond to the listed name in Figure 2(a). Extended Data Figure 3: digital images of the as-prepared flexible electrodes. CC represents the carbon cloth. CWM represents the MXene flakes wrapped carbon cloth. CPM represents the carbon cloth physically covered by a MXene shelter. Extended Data Figure 4: SEM image of (a) CC and (b) CWM electrode. Extended Data Figure 5: IR images of CC, CWM, CPM, and MXene film placed on a hot plate at different heating stages. The target temperature is shown in the upper-left corner. The plate temperature is shown in the lower-left corner. The time is displayed in the lower-right corner. The samples in the image are arranged in the same order as Extended Data Figure 3. Extended Data Figure 6: digital image of the flexible capacitor. Electrodes are attached to both sides of the hydrogel electrolyte. Extended Data Figure 7: digital image of the wearable textile before (left) and after (right) MXene wrap. Extended Data Table 1: IR emissivity reported by literatures.
