# Supplementary material for: Low Infrared Emissivity and Strong Stealth of Ti-Based MXenes
Source: Research (Wash D C). 2022 May 23;2022:9892628. doi: 10.34133/2022/9892628 (PMC9157363; doi:10.34133/2022/9892628)
Supplement: Supplementary Materials — Extended Data Figure 1: AFM image of Ti3C2TX film. Extended Data Figure 2: digital images of the reference samples that are physically covered by MXene film. The numbers correspond to the listed name in Figure 2(a). Extended Data Figure 3: digital images of the as-prepared flexible electrodes. CC represents the carbon cloth. CWM represents the MXene flakes wrapped carbon cloth. CPM represents the carbon cloth physically covered by a MXene shelter. Extended Data Figure 4: SEM image of (a) CC and (b) CWM electrode. Extended Data Figure 5: IR images of CC, CWM, CPM, and MXene film placed on a hot plate at different heating stages. The target temperature is shown in the upper-left corner. The plate temperature is shown in the lower-left corner. The time is displayed in the lower-right corner. The samples in the image are arranged in the same order as Extended Data Figure 3. Extended Data Figure 6: digital image of the flexible capacitor. Electrodes are attached to both sides of the hydrogel electrolyte. Extended Data Figure 7: digital image of the wearable textile before (left) and after (right) MXene wrap. Extended Data Table 1: IR emissivity reported by literatures. [file 9892628.f1.docx]

Supporting Information

Low infrared emissivity and strong stealth of Ti-based MXenes

Xinliang Li^1#^, Minghang Li^2#^, Xin LI^2^, Xiaomeng Fan^2^*, Chunyi Zhi^1^*

Correspondence to: [fanxiaomeng@nwpu.edu.cn](mailto:fanxiaomeng@nwpu.edu.cn); cy.zhi@cityu[.edu.hk](mailto:fanxiaomeng@nwpu.edu.cn)

X.L.L and M.H.L contributed equally to this work

**This PDF file includes:**

Materials and Methods

Supplementary Text

Figs. S1 to S7

Table S1

References (1-34)

Methods

**Materials.** Ti_3_AlC_2_, Ti_2_AlC, TiVAlC, MAX ceramic powders (Jilin 11 Technology Co., Ltd), lithium fluoride (LiF, Aladdin, 99%), hydrochloric acid (HCl, Aladdin, 37%), hydrofluoric acid (HF, Aladdin, 40 wt.%), zinc sulfate (ZnSO_4_, Aladdin), polyvinylidene fluoride (PVDF; AR grade, Aladdin) binder, Acrylamide (AM; electrophoresis grade, Aladdin), N-Methylpyrrolidone (NMP; AR grade, Aladdin), potassium persulfate (K_2_S_2_O_8_; AR grade, Aladdin) and 1 mg of N,N′-methylenebis (acrylamide) (MBAAm, chemical grade, Aladdin).

**Synthesis of MXene films.** Typically, flexible Ti_3_C_2_T_X_ MXene flakes were prepared using Ti_3_AlC_2_ MAX ceramic precursor and aqueous HCl+LiF etchant. First, the etchant solution was fabricated by dissolving 2 g of LiF powder into 40 ml of 9 M HCl solution, followed by vigorously stirred for 0.1 h. Then, 2g of Ti_3_AlC_2_ MAX was slowly dispersed into the as-prepared etchant solution. After that, the mixture was sealed and vigorously stirred at 35 ºC for 48 h. Next, the slurry was filtered and washed the sediment with deionized water by centrifuging until the pH of the suspension was close to 7. The sediment was then repeatedly centrifuged with non-recycling deionized water until the suspension was cloudy, where the rotational speed was set to 3500 rpm for each period of 5 minutes. The turbid suspension was the required aqueous MXene dispersion. Finally, the dispersion concentration was estimated by weighting the film mass. Similarly, Ti_2_CT_X_ MXene flakes were prepared using the same process described above, but the stirring time and temperature were set to 24 h and 30 ºC. For TiVCT_x_ MXene, the etching time and temperature were set to 60 h and 55 ºC. The films were obtained by a facile vacuum method, and their thicknesses were controlled by tuning the applied volume of MXene dispersion.

**Synthesis of MXene wrapped carbon cloth and textile.** The flexible MXene wrapped carbon cloth and textile were fabricated by a facile self-assembly approach. Typically, the carbon cloth and textile were placed directly into MXene dispersion and stirred vigorously, during which MXene flakes spontaneously coated the carbon fibers or textile fiber inside. After being dried in a vacuum oven at room temperature, the required MXene-functionalized carbon cloth and textile were obtained.

**Synthesis of wearable quasi-solid capacitor.** The MXene wrapped carbon cloth electrodes for the quasi-solid capacitor were prepared by simply mixing active carbon with PVDF in NMP solvent with a mass ratio of 80:20 and stirred vigorously for 0.5 h. Then, the wet slurry was cast on the above MXene-functionalized carbon cloth, followed by drying at 70 ºC for 48 h in a vacuum oven. Then the quasi-solid electrolyte was synthesized. Specifically, 1g of acrylamide monomer powders was added into 10 ml of deionized water and vigorously stirred at room temperature for 0.5 h. 5 mg of potassium persulfate (initiator) and 1 mg of N,N′-methylenebis(acrylamide) were then dispersed into the aqueous solution, followed by vigorously stirred for 25 °C for 2 h. Then, the homogeneous solution was injected into the homemade glass mold and heated at 50 °C for 4 h to realize the polymerization reaction. Next, the obtained hydrogel was soaked in an aqueous electrolyte solution of 2 M ZnSO_4_ over 48 h for realizing the ions exchange equilibration. Finally, the full capacitor was assembled by directly attaching the as-prepared electrode to both sides of the electrolyte.

**Materials Characterization.** A scanning electron microscope (SEM; S-4700, Hitachi) was employed to characterize the microstructure and morphology. X-ray diffractometer equipment (XRD; Bruker, D2 Avance) was carried out to collect the XRD pattern for analysing the phase composition. Atomic force microscope image was recorded with a Dimension FastScan AFM equipment (AFM; Bruker)

**Infrared emissivity measurement.** Two measurements, direct active blackbody radiation source method (2-20 μm) and indirect Fourier transform infrared spectroscopy technique, were performed to characterize the infrared emissivity. Infrared emissivity tester (TSS-5X, Japan) and Fourier transform infrared (FTIR, Thermo, Nicolet6700, USA) equipment were employed. A thermal imaging camera (UTi220A; China) was used to capture the infrared images. For direct active blackbody radiation source method, MXene film used was shaped into a circle shape with the diameter of 47 mm. For indirect Fourier transform infrared spectroscopy technique, MXene film was coated on a quartz glass sheet with a size of 22.86 mm x 10.16 mm.


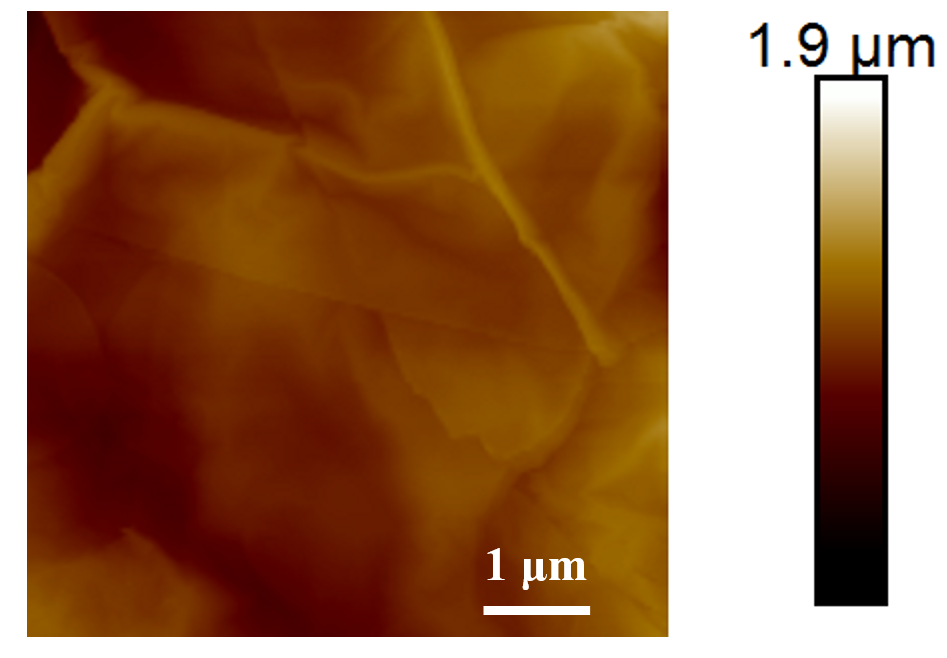


**Extended Data Fig. 1 |** AFM image of Ti_3_C_2_T_X_ film.


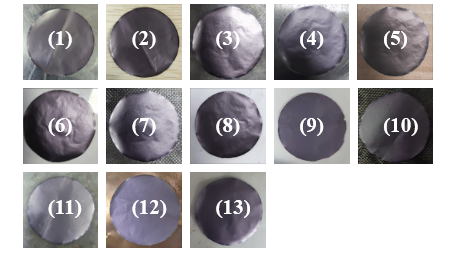


**Extended Data Fig. 2 |** Digital images of the reference samples that are physically covered by MXene film. The numbers correspond to the listed name in Fig. 2a.


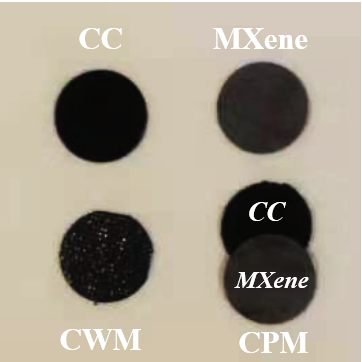


**Extended Data Fig. 3 |** **Digital images of the as-prepared flexible electrodes. CC represents the carbon cloth. CWM represents the MXene flakes wrapped carbon cloth. CPM represents the carbon cloth physically covered by a MXene shelter.**


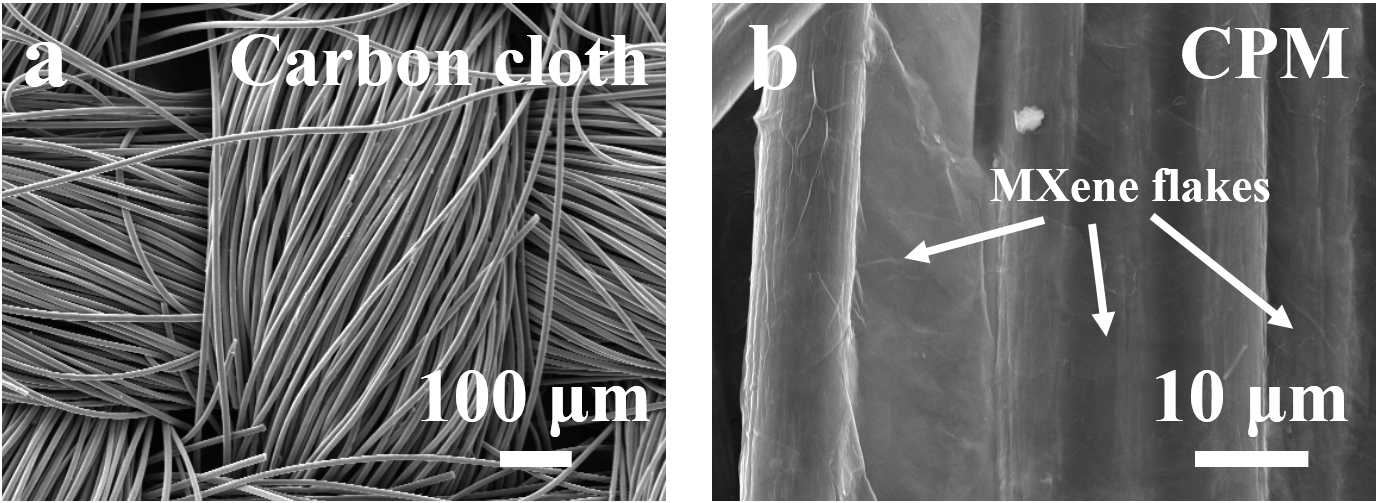


**Extended Data Fig. 4 |** SEM image of **a,** CC and **b,** CWM electrode.


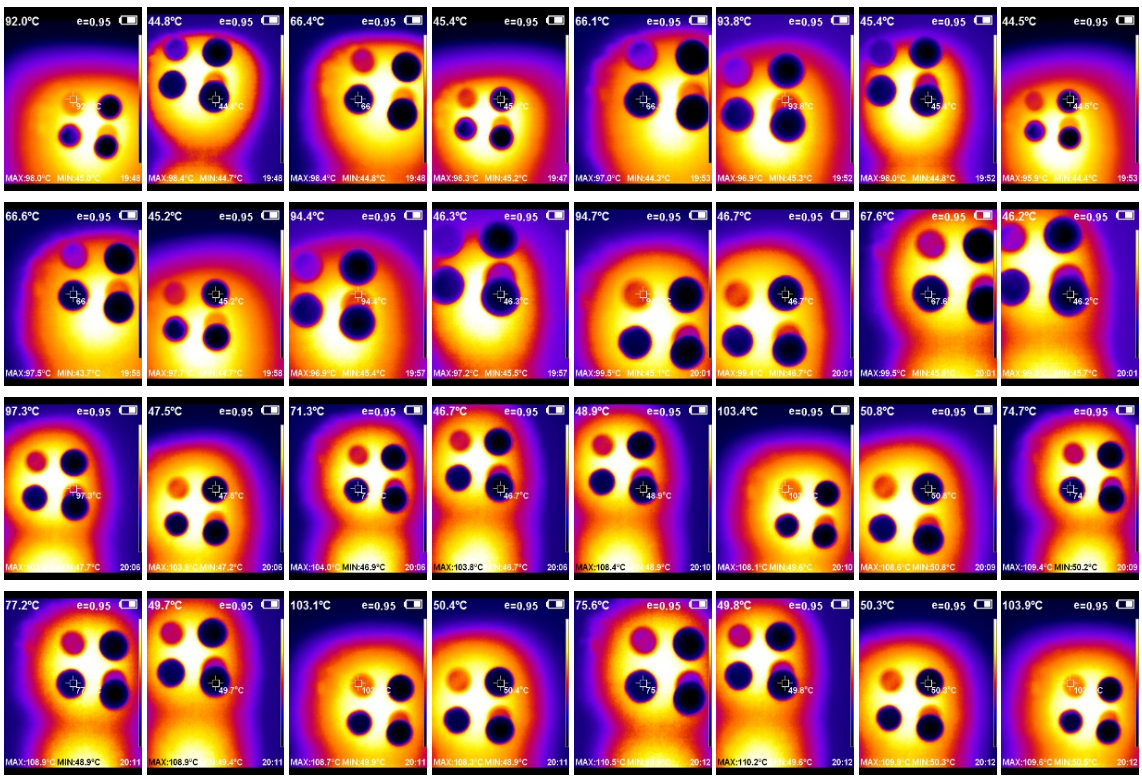


**Extended Data Fig. 5 |**. **IR images of CC, CWM, CPM, MXene film placed on a hot plate at different heating stages. The target temperature is shown in the upper-left corner. The plate temperature is shown in the lower-left corner. The time is displayed in the lower-right corner. The samples in the image are arranged in the same order as Fig. S2 above.**


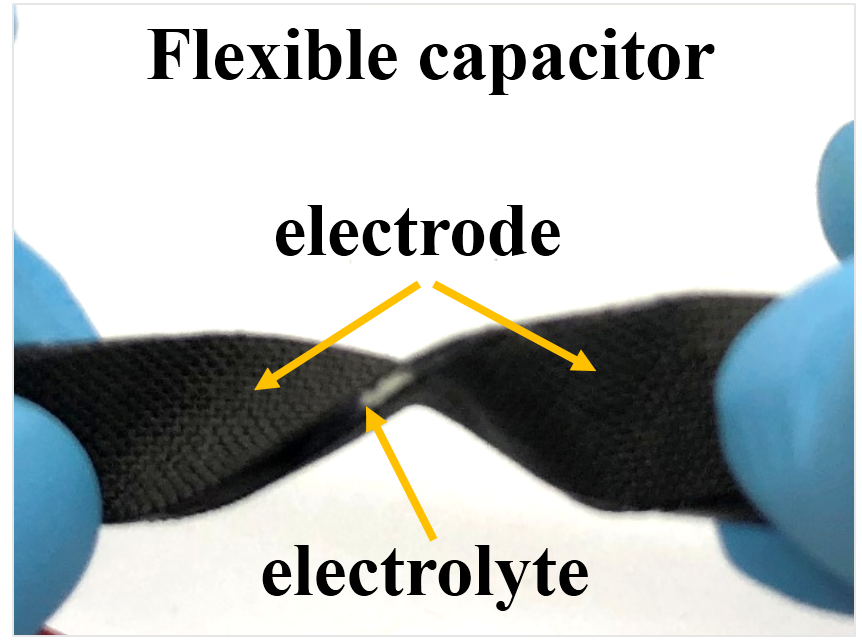


**Extended Data Fig. 6 |** **Digital image of the flexible capacitor. Electrodes are attached to both sides of the hydrogel electrolyte.**


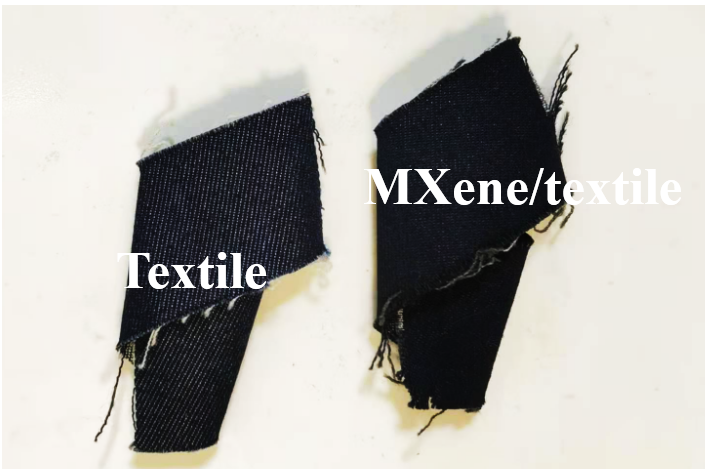


**Extended Data Fig. 7 | Digital image of the wearable textile before (left) and after MXene wrap (right).**

**Extended Data Table. 1 | IR emissivity reported by literatures.**

| **Sample** | **Thickness (μm)** | **IR Band**  **(μm)** | **IR emissivity** | **Ref** |
| --- | --- | --- | --- | --- |
| **Ti_3_C_2_T_X_** | **5** | **2-22** | **0.18** | **This work** |
| **Ti_2_CT_X_** | **5** | **2-22** | **0.22 ± 0.01** | **This work** |
| **TiVCT_X_** | **5** | **2-22** | **0.20 ± 0.01** | **This work** |
| Metasurface | 350 | 8-14 | 0.49 | (*1*) |
| CNT film array | 22 | 7.5-13 | 0.15 | (*2*) |
| crumpled graphene | 10 | 7.5-15 | 0.50 | (*3*) |
| graphite | 16 | 5.5-17 | 0.72 | (*4*) |
| Al | 1000 | 2.5-25 | 0.39 | (*5*) |
| Al flakes | 2000 | None | 0.39 | (*6*) |
| Al powder | 90 | 8-14 | 0.60 | (*7*) |
| Ag powder | 60 | 8–14 | 0.14 | (*8*) |
| Al/Ni core–shell pigments | 60 | 8-14 | 0.46 | (*9*) |
| Ge/ZnS photonic crystal | 3.5 | 3-5.5 | 0.10 | (*10*) |
| SiO_2_–ZnO photonic crystal | 5 | 3-5.5 | 0.45 | (*11*) |
| Al_2_O_3_ | 1000 | 8-20 | 0.85 | (*12*) |
| WO_3_.H2O | 100 | 2-22 | 0.60 | (*13*) |
| ZnO | 400 | None | 0.38 | (*14*) |
| ZnO | 600 | None | 0.47 | (*14*) |
| Al-doped SrZrO_3_ | 2000 | 3-5 | 0.50 | (*15*) |
| SrZrO_3_ | 2000 | 3-5 | 0.668 | (*15*) |
| Indium oxide | 12 | 8-14 | 0.91 | (*16*) |
| Al_2_O_3_-Al 6061 | 16 | 10–16 | 0.90 | (*17*) |
| W-doped VO_2_ | 600 | 8-14 | 0.75 | (*18*) |
| PU | 300 | 2-22 | 0.83 | (*19*) |
| Collagen | >10 | 8-14 | 0.851 | (*16*) |
| PANI film | >20 | 3-5 | 0.56 | (*20*) |
| WPU | 10000 | 2-22 | 0.94 | (*21*) |
| HClO_4_ doped-PANI | 20 | 2.5-25 | 0.42 | (*22*) |
| ATO/PAN-xVO_2_ | 0.2 | 2-20 | 0.82 | (*23*) |
| PU/Al/AAA | 120 | 2.5-25 | 0.70 | (*24*) |
| [(CNT/Al/PA_6_)/PU]_3_-PVA/SiO_2_-PU | 930 | 2-22 | 0.71 | (*19*) |
| PVA-SIO_2_ | 300 | 2-22 | 0.84 | (*19*) |
| Al–SiO_2_ nanoparticle composite | 0.2 | 3-14 | 0.12 | (*25*) |
| Al pigment -graphene | 50 | 8-14 | 0.68 | (*26*) |
| Al/MnO_2_ composite pigments | 80 | 8-14 | 0.30 | (*27*) |
| Al/Fe_3_O_4_ core–shell composite | 50 | 8-14 | 0.33 | (*28*) |
| Al/Co_3_O_4_ composite pigments | 50 | 8-14 | 0.44 | (*6*) |
| collagen-g-PMMA/indium oxide | >10 | 8-14 | 0.58 | (*16*) |
| PDMS/PU/Ag-SiO_2_ | 60 | 8–14 | 0.83 | (*8*) |
| Acrylic/Al | 50 | 8-13 | 0.35 | (*29*) |
| EPDM/Cu | 50 | 3-25 | 0.10 | (*30*) |
| WAX-al | 90 | 8-14 | 0.78 | (*7*) |
| EPDM/Cu | 40 | 8-14 | 0.10 | (*31*) |
| Al powder into epoxy-siloxane | 20 | 8-14 | 0.23 | (*32*) |
| Al-PANI | 2 | 8-14 | 0.55 | (*5*) |
| WPU-silver-plated hollow glass microsphere | 10000 | 2-22 | 0.71 | (*21*) |
| acrylic resin/flake copper | 21 | 3-5 | 0.61 | (*33*) |
| PDMS modified polyurethane/Ag | 60 | 8–14 | 0.14 | (*33*) |
| PU/Cu | 40 | 8-14 | 0.45 | (*34*) |
| Al/PANI | 1000 | 2.5-25 | 0.55 | (*5*) |

**References**

1. Y. Ma *et al.*, A transparent and flexible metasurface with both low infrared emission and broadband microwave absorption. *Journal of Materials Science-Materials in Electronics* **32**, 2001-2010 (2021).

2. Y. Sun *et al.*, Large‐Scale Multifunctional Carbon Nanotube Thin Film as Effective Mid‐Infrared Radiation Modulator with Long‐Term Stability. *Advanced Optical Materials* **9**, (2020).

3. A. Krishna *et al.*, Ultraviolet to Mid-Infrared Emissivity Control by Mechanically Reconfigurable Graphene. *Nano Lett* **19**, 5086-5092 (2019).

4. F. Wang, L. Cheng, H. Mei, Q. Zhang, L. Zhang, Effect of Surface Microstructures on the Infrared Emissivity of Graphite. *International Journal of Thermophysics* **35**, 62-75 (2014).

5. A. Zhu, H. Xing, Q. Fan, X. Ji, P. Yang, Conductive polyaniline coated on aluminum substrate as bi-functional materials with high-performance microwave absorption and low infrared emissivity. *Synthetic Metals* **271**, (2021).

6. Y. Liu *et al.*, The synthesis and characterization of Al/Co 3 O 4 magnetic composite pigments with low infrared emissivity and low lightness. *Infrared Physics & Technology* **83**, 88-93 (2017).

7. G. Wu, D. Yu, Preparation and characterization of a new low infrared-emissivity coating based on modified aluminum. *Progress in Organic Coatings* **76**, 107-112 (2013).

8. E. E. Ateia, M. Abd Elhay Metwaly, H. R. Tantawy, Preparation and properties of novel infrared low-emissive coating of acrylic resin/flake copper composites”. *Materials Research Innovations*, 1-7 (2021).

9. L. Yuan, J. Hu, X. Weng, Q. Zhang, L. Deng, Galvanic displacement synthesis of Al/Ni core–shell pigments and their low infrared emissivity application. *Journal of Alloys and Compounds* **670**, 275-280 (2016).

10. W. Zhang, G. Xu, X. Shi, H. Ma, L. Li, Ultra-low infrared emissivity at the wavelength of 3–5μm from Ge/ZnS one-dimensional photonic crystal. *Photonics and Nanostructures - Fundamentals and Applications* **14**, 46-51 (2015).

11. Y. Yang *et al.*, The compatible performance of three-dimensional SiO2–ZnO amorphous photonic crystals in adjustable structural color and low infrared emissivity. *Optical Materials* **107**, (2020).

12. Y. M. Wang *et al.*, An elevated temperature infrared emissivity ceramic coating formed on 2024 aluminium alloy by microarc oxidation. *Ceramics International* **39**, 2869-2875 (2013).

13. A. Bessiere *et al.*, Flexible electrochromic reflectance device based on tungsten oxide for infrared emissivity control. *Journal of Applied Physics* **91**, 1589-1594 (2002).

14. L. M. Marques, S. Braz, E. C. Fernandes, High emissivity ZnO coatings prepared from chloride electrolyte by electrodeposition on a “Dynamic Hydrogen Bubble Template”. *Infrared Physics & Technology* **113**, (2021).

15. E. Li *et al.*, Infrared radiation and thermal properties of Al-doped SrZrO3 perovskites for potential infrared stealth coating materials in the high-temperature environment. *Ceramics International*, (2021).

16. Y. Shan *et al.*, Preparation and infrared emissivity study of collagen-g-PMMA/In2O3 nanocomposite. *Materials Letters* **58**, 1655-1660 (2004).

17. M. M. S. Al Bosta, K.-J. Ma, H.-H. Chien, The effect of MAO processing time on surface properties and low temperature infrared emissivity of ceramic coating on aluminium 6061 alloy. *Infrared Physics & Technology* **60**, 323-334 (2013).

18. Z. Mao *et al.*, Infrared stealth property based on semiconductor (M)-to-metallic (R) phase transition characteristics of W-doped VO2 thin films coated on cotton fabrics. *Thin Solid Films* **558**, 208-214 (2014).

19. K. Y. Fang, Y. C. Zhao, F. Fang, Infrared stealth nanofibrous composite with thermal stability and mechanical flexibility. *Journal of Alloys and Compounds* **855**, (2021).

20. F. F. Lu, P. Y. Tan, Y. G. Han, Variable infrared emissivity based on polyaniline electrochromic device influenced by porous substrate. *Journal of Applied Polymer Science* **138**, (2021).

21. Y. Chang, Y. Wang, W. Wang, D. Yu, Highly efficient infrared stealth asymmetric-structure waterborne polyurethane composites prepared via one-step density-driven filler separation method. *Colloids and Surfaces A: Physicochemical and Engineering Aspects* **614**, (2021).

22. L. Zhang *et al.*, Further understanding of the mechanisms of electrochromic devices with variable infrared emissivity based on polyaniline conducting polymers. *Journal of Materials Chemistry C* **7**, 9878-9891 (2019).

23. K. Y. Fang, Y. J. Wang, Y. C. Zhao, F. Fang, Infrared stealth nanofibrous composites with thermal adaptability and mechanical flexibility. *Composites Science and Technology* **201**, (2021).

24. Z. Ma *et al.*, Effects of Al-based alloy powders on the mechanical behavior, corrosion resistance and infrared emissivity of polyurethane composite coatings. *Colloids and Surfaces A: Physicochemical and Engineering Aspects* **624**, (2021).

25. L. Chen, Z. Ren, X. Liu, K. Wang, Q. Wang, Infrared–visible compatible stealth based on Al-SiO2 nanoparticle composite film. *Optics Communications* **482**, (2021).

26. K. Wang, C. Wang, Y. Yin, K. Chen, Modification of Al pigment with graphene for infrared/visual stealth compatible fabric coating. *Journal of Alloys and Compounds* **690**, 741-748 (2017).

27. Y. Liu *et al.*, The synthesis and optical properties of Al/MnO 2 composite pigments by ball-milling for low infrared emissivity and low lightness. *Progress in Organic Coatings* **108**, 30-35 (2017).

28. L. Yuan, X. Weng, J. Xie, W. Du, L. Deng, Solvothermal synthesis and visible/infrared optical properties of Al/Fe3O4 core–shell magnetic composite pigments. *Journal of Alloys and Compounds* **580**, 108-113 (2013).

29. L. Yuan, X. Weng, L. Deng, Influence of binder viscosity on the control of infrared emissivity in low emissivity coating. *Infrared Physics & Technology* **56**, 25-29 (2013).

30. H. Yu *et al.*, Effects of size, shape and floatage of Cu particles on the low infrared emissivity coatings. *Progress in Organic Coatings* **66**, 161-166 (2009).

31. H. Yu *et al.*, Preparation of leafing Cu and its application in low infrared emissivity coatings. *Journal of Alloys and Compounds* **484**, 395-399 (2009).

32. C. Hu, G. Xu, X. Shen, C. Shao, X. Yan, The epoxy-siloxane/Al composite coatings with low infrared emissivity for high temperature applications. *Applied Surface Science* **256**, 3459-3463 (2010).

33. H. P. Zhou *et al.*, Difunctional composite coatings with low infrared emissivity and electrostatic dissipation property. *Infrared Physics & Technology* **113**, (2021).

34. H. Yu, G. Xu, X. Shen, X. Yan, C. Cheng, Low infrared emissivity of polyurethane/Cu composite coatings. *Applied Surface Science* **255**, 6077-6081 (2009).
